# Misfolded α-synuclein causes hyperactive respiration without functional deficit in live neuroblastoma cells

**DOI:** 10.1242/dmm.040899

**Published:** 2020-01-17

**Authors:** Cathryn L. Ugalde, Sarah J. Annesley, Shane E. Gordon, Katelyn Mroczek, Matthew A. Perugini, Victoria A. Lawson, Paul R. Fisher, David I. Finkelstein, Andrew F. Hill

**Affiliations:** 1 Department of Biochemistry and Genetics, La Trobe University, Bundoora, VIC 3086, Australia; 2 Department of Microbiology and Immunology, University of Melbourne, Parkville, VIC 3052, Australia; 3 Howard Florey Institute of Neuroscience and Mental Health, Parkville, VIC 3052, Australia; 4 Department of Biochemistry and Molecular Biology, University of Melbourne, Parkville, VIC 3052, Australia; 5 Department of Physiology, Anatomy and Microbiology, La Trobe University, Bundoora, VIC 3086, Australia; 6 La Trobe University-Comprehensive Proteomics Platform, La Trobe University, Bundoora, VIC 3086, Australia

**Keywords:** α-Synuclein, Synucleinopathy, Parkinson's disease, Mitochondria, Cardiolipin, PMCA

## Abstract

The misfolding and aggregation of the largely disordered protein, α-synuclein, is a central pathogenic event that occurs in the synucleinopathies, a group of neurodegenerative disorders that includes Parkinson's disease. While there is a clear link between protein misfolding and neuronal vulnerability, the precise pathogenic mechanisms employed by disease-associated α-synuclein are unresolved. Here, we studied the pathogenicity of misfolded α-synuclein produced using the protein misfolding cyclic amplification (PMCA) assay. To do this, previous published methods were adapted to allow PMCA-induced protein fibrillization to occur under non-toxic conditions. Insight into potential intracellular targets of misfolded α-synuclein was obtained using an unbiased lipid screen of 15 biologically relevant lipids that identified cardiolipin (CA) as a potential binding partner for PMCA-generated misfolded α-synuclein. To investigate whether such an interaction can impact the properties of α-synuclein misfolding, protein fibrillization was carried out in the presence of the lipid. We show that CA both accelerates the rate of α-synuclein fibrillization and produces species that harbour enhanced resistance to proteolysis. Because CA is virtually exclusively expressed in the inner mitochondrial membrane, we then assessed the ability of these misfolded species to alter mitochondrial respiration in live non-transgenic SH-SY5Y neuroblastoma cells. Extensive analysis revealed that misfolded α-synuclein causes hyperactive mitochondrial respiration without causing any functional deficit. These data give strong support for the mitochondrion as a target for misfolded α-synuclein and reveal persistent, hyperactive respiration as a potential upstream pathogenic event associated with the synucleinopathies.

This article has an associated First Person interview with the first author of the paper.

## Abstract (precis)

**Summary:** Misfolded α-synuclein produced using the protein misfolding cyclic amplification assay associates with cardiolipin and causes hyperactive respiration in neuronal cells.

## INTRODUCTION

Parkinson's disease (PD) is a slowly progressing movement disorder clinically defined by the appearance of bradykinesia, rigidity and/or tremor. Pathological features of PD are the appearance of Lewy bodies, which are intracellular protein deposits predominately composed of misfolded α-synuclein and neuronal loss. PD is one of a group of neurodegenerative conditions called synucleinopathies that have in common the aggregation of misfolded α-synuclein. The profiles of neuronal vulnerability and protein deposition within neurons and glial cells vary amongst the conditions (Galvin et al., 2001; Surmeier et al., 2017), and PD is associated with the progressive loss of dopaminergic neurons in the substantia nigra pars compacta.

Largely an unstructured protein that is localized to the synapse under normal physiological conditions, α-synuclein, in its misfolded form, is strongly linked to the development of disease. This is evidenced by the finding that various point mutations in the gene encoding α-synuclein, *SNCA* (such as A53T), cause early-onset familial disease (Appel-Cresswell et al., 2013; Krüger et al., 1998; Lesage et al., 2013; Polymeropoulos et al., 1997; Proukakis et al., 2013; Zarranz et al., 2004), as do gene duplications (Chartier-Harlin et al., 2004) and triplications (Singleton et al., 2003). Likewise, there is also now a large body of data showing the ability of α-synuclein to adopt pathogenic properties upon misfolding (Ugalde et al., 2016).

Despite the numerous data describing the pathogenicity of misfolded α-synuclein, the precise molecular mechanisms that α-synuclein employs that contribute to the pathogenesis of the synucleinopathies is currently unclear. A consideration to studying these disorders is the complex nature of α-synuclein misfolding; α-synuclein is an amyloid-forming protein and thus can adopt numerous misfolded conformations. Precursors to the mature fibrils abundant in Lewy bodies, small soluble oligomers and protofibrils are also present in the brain in PD (Garcia-Esparcia et al., 2015; Sharon et al., 2003; Tofaris et al., 2003), and, critically, oligomers are considered to be the most toxic conformation (Ugalde et al., 2016). The process of α-synuclein fibril formation sees these highly dynamic species exist as part of a heterogeneous mix of various-sized conformations that maintain a state of equilibrium and, through interspecies interactions, can both expand and contract to higher- or lower-ordered structures (Cremades et al., 2012; Roberts and Brown, 2015).

Many studies examining the properties of pathogenic α-synuclein induce misfolding in bacterially expressed recombinant protein to produce a homogenous population of a defined misfolded structure. Although these common techniques of fibrillization are useful to attribute pathogenic properties to a specific conformation, they are not reflective of the numerous conformations of pathogenic α-synuclein that exist *in vivo*. A novel fibrillization technique that may alleviate these issues is protein misfolding cyclic amplification (PMCA). PMCA was originally developed to study the generation of prions (Saborio et al., 2001), which are proteinaceous and transmissible neurodegenerative agents primarily composed of PrP^Sc^, a misfolded form of the normal protein, PrP^C^ (also known as PRNP). Recently, PMCA has been shown capable of producing misfolded forms of α-synuclein that appear to encompass various conformations, including oligomers (Herva et al., 2014). Current studies on the pathogenicity of α-synuclein species formed via PMCA are limited to low concentrations of dilute protein due to a high concentration of detergent in the conversion buffer. As such, we sought to study the properties of PMCA-generated α-synuclein by adapting published methods to produce misfolded species under non-toxic conditions and extensively characterizing formed species. Next, to gain insight into the pathogenic properties of misfolded α-synuclein, an unbiased lipid screen of 15 biologically relevant lipids was performed. We show that PMCA-generated α-synuclein associates with cardiolipin (CA), and further assessment revealed that this lipid can both accelerate fibrillization and modify the biophysical and biochemical properties of α-synuclein species formed by enhancing their resistance to proteolysis and increasing insoluble load. Given that CA is a lipid almost exclusively expressed in the inner mitochondrial membrane (IMM), we then sought to determine whether this interaction has relevance to cellular functioning by assessing the ability of PMCA-generated α-synuclein to modulate mitochondrial respiration in live non-transgenic neuroblastoma cells. Comprehensive analysis using the Seahorse XFe24 Analyzer showed that misfolded α-synuclein causes hyperactive mitochondrial respiration without causing dysfunctional activity at any complex. This also occurred independently of upstream metabolic changes in glycolysis. These rigorous data in cells with normal endogenous protein suggest that the mitochondrion is a target for misfolded α-synuclein and that hyperactive respiration may be an early, upstream pathogenic process that neuronal cells experience in the synucleinopathy disorders.

## RESULTS

### PMCA can produce misfolded α-synuclein species in prion conversion buffer

**Fig. 1.**
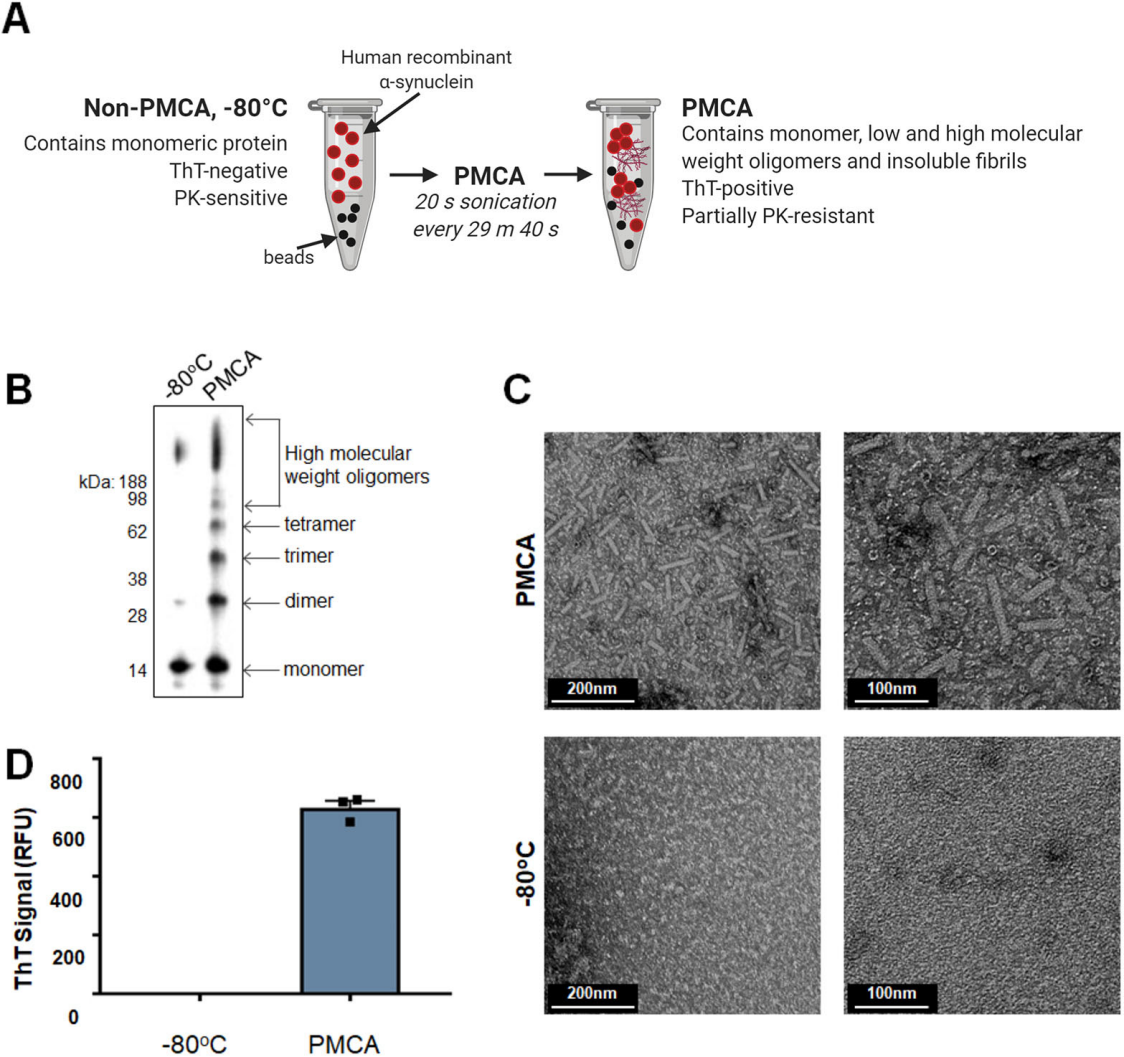
**α-synuclein PMCA.** (A) Workflow of α-synuclein PMCA: lyophilized recombinant wild-type protein was reconstituted in buffer to 90 µM and 60 µl was aliquoted into PCR tubes. Samples were exposed to PMCA in the presence of beads. Sonications were set to occur for 20 s every 29 min 40 s for a total process time of 72 h. This figure was created with Biorender.com. (B–D) The extent of fibrillization in protein exposed to PMCA and non-PMCA controls (−80°C) was assessed by (B) western immunoblot analysis using α-synuclein-specific monoclonal antibody MJFR1 (amino acid specificity: 118–123), (C) transmission electron microscopy (TEM) (representative images shown) and (D) thioflavin T (ThT) fluorescence, where the values obtained for each experimental replicate represented the average fluorescence of triplicate wells after subtraction of a blank well to account for background fluorescence. RFU, relative fluorescent units. Data are presented as mean±s.e.m. (*n*=3) and represent every experimental replicate performed.

Generating misfolded α-synuclein species using PMCA was first developed following the protocol outlined by Herva et al. (2014) (Fig. 1A). Because the buffer used by Herva et al. to dissolve α-synuclein is routinely used as the conversion buffer for prion PMCA reactions, it is hereafter referred to as prion conversion buffer (PCB). The extent of fibrillization of α-synuclein in PCB following exposure to PMCA for 24 h was assessed using western immunoblotting, transmission electron microscopy (TEM) and thioflavin T (ThT) fluorescence. Here, western immunoblotting was employed to identify small conformations of misfolded α-synuclein that may be resolved by SDS-PAGE, and showed that samples subjected to PMCA contained a range of various-sized species, with monomeric protein observed at 14 kDa and dimer, trimer and tetramer oligomers identified by their increase in molecular mass by intervals of the weight of the monomer (Fig. 1B). Species greater than a tetramer, separated by the size of one monomeric unit, were identified all the way up to the high-molecular-mass range of the gel. In comparison, a non-PMCA sample stored at −80°C during the PMCA process was almost entirely composed of monomeric protein (Fig. 1B). TEM was next used to confirm the presence of large mature α-synuclein fibrils. Consistent with the TEM images reported by Herva et al. (2014), PMCA-generated α-synuclein fibrils were found to be rod-like in structure. No aggregates were observed in the −80°C sample (Fig. 1C). The compound ThT exhibits fluorescence upon binding to β-sheet structures (Biancalana and Koide, 2010; Vassar and Culling, 1959) and hence is universally used to detect amyloid. Upon exposure to 24 h PMCA, α-synuclein adopted strong ThT reactivity, while the monomeric −80°C sample displayed no fluorescent signal (Fig. 1D). Collectively, these data confirm that PMCA is a useful tool to produce misfolded α-synuclein.

### PMCA-induced misfolding of α-synuclein can occur under non-toxic conditions

Because the PCB used to reconstitute protein in Fig. 1 contains Triton X-100, a limitation to studying the pathogenicity of PMCA-generated α-synuclein is the inherent toxicity of the buffer. Accordingly, in order to progress to studies on the pathogenicity of PMCA-generated α-synuclein, it was necessary to isolate these species in a buffer that did not contain detergent. Given the small size and highly dynamic nature of oligomers, buffer exchange or centrifugation were not considered viable options to collect or maintain the integrity of small, oligomeric species that may be produced in this system. Instead, the replacement of the conversion buffer, PCB, with a non-toxic analogue was pursued. In order to address this, the extent of PMCA-induced fibrillization of α-synuclein was assessed following its reconstitution in several buffers assumed to be non-toxic. The following buffers were tested: PBS, PBS+150 mM NaCl (PBSN), TBS and TBS+150 mM NaCl (TBSN). These samples were exposed to 24 h PMCA as per the same conditions detailed in Fig. 1 and were compared to an equivalent sample of α-synuclein dissolved in PCB buffer (α-synuclein:PCB). Western immunoblotting and ThT were used as readouts to compare the extent of α-synuclein fibrillization in the various buffers (Fig. S1). The ThT data showed that, following PMCA, all buffers were able to produce species that contained ThT-reactive conformations (Fig. S1A). Resolving samples on SDS-PAGE and immunodetection of α-synuclein gave further information on the α-synuclein species produced by the buffers (Fig. S1B). This assay revealed differences in the efficiency of the buffers to induce fibrillization, with PCB clearly being the most proficient buffer for producing oligomeric species. The degree of fibrillization was variable amongst the non-toxic buffers, with PBSN being the most effective non-toxic buffer for α-synuclein misfolding. This observation is consistent with studies showing that salt concentrations are positively associated with α-synuclein fibrillization (Hoyer et al., 2002).

### Characterization of PMCA-generated α-synuclein misfolding in PBSN

**Fig. 2.**
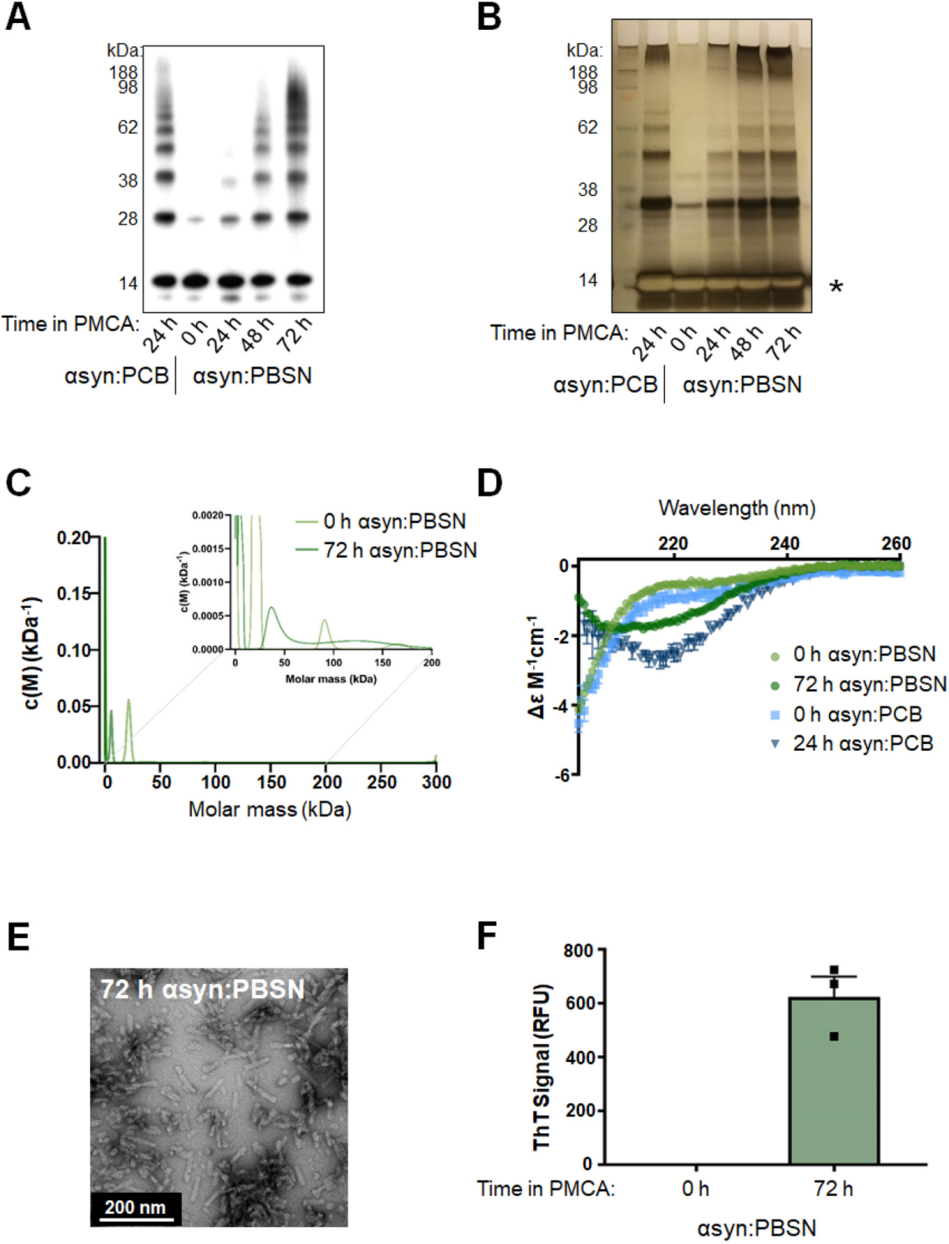
**Production of misfolded α-synuclein species in PBSN buffer produces comparable misfolded content to α-synuclein species generated in PCB.** Lyophilized recombinant wild-type protein was reconstituted in PBSN (α-synuclein:PBSN) or PCB (α-synuclein:PCB) and subjected to PMCA for a total process time of 0, 24, 48 or 72 h. (A,B) Extent of fibrillization in samples was assessed using (A) western immunoblot analysis using α-synuclein-specific monoclonal antibody MJFR1 (amino acid specificity: 118–123) and (B) silver staining, where the asterisk indicates saturation of the protein signal. (C) Analytical ultracentrifugation sedimentation velocity analyses of non-PMCA and PMCA-generated α-synuclein. Continuous mass distribution [*c*(*M*)] distributions are shown as a function of molar mass. Inset shows zoomed-in image of values between 0 and 200 kDa. Best fits to experimental data yielded a root mean square deviation of 0.0055 and 0.0045, *f*/*f*_0_ of 2.2 and 1.1, and Runs Test Z of 12 and 0.59 for non-PMCA and PMCA-generated α-synuclein, respectively. (D) Secondary structure of non-PMCA (0 h) or PMCA-generated (24 h or 72 h) α-synuclein:PBSN and α-synuclein:PCB was determined using circular dichroism (CD) spectroscopy (*n*=1, representative spectra). (E) TEM of fibrils produced in PBSN after 72 h PMCA (representative image). (F) α-Synuclein:PBSN exposed to PMCA for 72 h or non-PMCA (0 h) monomeric controls were further characterized by ThT fluorescence, where the values obtained for each experimental replicate represented the average fluorescence of triplicate wells after subtraction of a blank well to account for background fluorescence. RFU, relative fluorescent units. Data are presented as mean±s.e.m. (*n*=3) and represent every experimental replicate performed.

Given that none of the non-toxic buffers were as effective as PCB at facilitating misfolding of α-synuclein based on western immunoblotting, the protocol was further adapted with the most efficient non-toxic buffer, PBSN (Fig. 2). To assess this, the total process time of 24 h was extended to determine whether PBSN can produce comparable misfolded α-synuclein content to PCB if exposed to PMCA for a longer period. To this end, an incremental time-course experiment was performed, in which α-synuclein:PBSN was subjected to PMCA for a total process time of 0, 24, 48 and 72 h. These samples were compared to α-synuclein:PCB exposed to 24 h PMCA. Western immunoblot analysis revealed that the abundance of misfolded species produced in PBSN continues to amplify beyond a 24-h PMCA process time, with time-dependent elevations in misfolded content observed in samples exposed to PMCA for 48 h and 72 h. Following exposure to PMCA for 72 h, the presence of oligomeric species in α-synuclein:PBSN was comparable to that in 24-h α-synuclein:PCB (Fig. 2A). Because the use of western immunoblotting to detect misfolded α-synuclein can be compromised by artefacts associated with antibody epitope recognition, silver staining was also employed to detect total protein. Using this method confirmed that PMCA induces the generation of α-synuclein oligomers that are time dependent (Fig. 2B). However, unlike the western immunoblotting data (Fig. 2A), saturation was observed in the monomeric band (Fig. 2B; indicated by an asterisk). Collectively, these data show that although PMCA is capable of producing α-synuclein oligomers, the unsaturated signals from the western immunoblot analysis may not represent the exact ratios of species that resolve through SDS-PAGE, likely related to antibody epitope specificity.

Following on from these findings, sedimentation velocity experiments were next conducted using analytical ultracentrifugation (AUC) to provide an in-solution assessment of the presence of oligomeric species in PMCA-generated α-synuclein. In order to align with the experimental procedure of other assays, these experiments were conducted at 25°C and 37°C for non-PMCA (0 h α-synuclein:PBSN) and PMCA-generated α-synuclein (72 h α-synuclein:PBSN), respectively. Continuous mass distribution profiles [*c*(*M*)] were given for both proteins by fitting sedimentation profiles to the *c*(*M*) distribution model (Schuck, 2000; Schuck et al., 2002). As shown in Fig. 2C, both distributions show a single predominant peak of mass 6–20 kDa that is a reasonable approximation of the α-synuclein monomer (≈14 kDa). A best-fit, weight-average *f*/*f*_0_ value of 2.2 was found for the non-PMCA α-synuclein sample, indicating a very prolate solution conformation; contrastingly the *f*/*f*_0_ for PMCA-treated α-synuclein was 1.1, consistent with a more globular shape. Closer inspection of the region spanning 0–200 kDa (Fig. 2C, inset) reveals the presence of several broad peaks between 20 kDa and 60 kDa and 100 kDa and 150 kDa. Compellingly, the molecular assignments for these peaks are in good general agreement with the western immunoblotting and silver staining data for the equivalent samples. Residuals for fits to *c*(*M*) distributions appear random and non-systematic, indicating a good fit of data to the models (Fig. S2B). Thus, these data support the presence of low-molecular-mass oligomers in solution in PMCA-generated α-synuclein:PBSN.

For the AUC data acquisition, a higher rotor speed of 42,000 rpm was selected to maximize resolution of low-molecular-mass components. To assess the extent to which depletion of high sedimentation coefficient solutes (e.g. fibrils) occurs, we compared absorbance-detected sedimentation profiles used for *c*(*M*) distribution models (collected at 42,000 rpm) with those collected at a lower rotor speed (3000 rpm), at which sedimentation of these larger components is expected to be minimal (Fig. S2A). An approximate sixfold loss of absorbance intensity is apparent after acceleration to the higher rotor speed of 42,000 rpm, indicating rapid sedimentation of solutes with high sedimentation coefficients (Fig. S2A). This is further support of an expansion in fibrillar content with PMCA treatment. Contrastingly, under these conditions we find negligible loss of absorbance signal for non-PMCA α-synuclein.

Having characterized misfolded protein using AUC, further assessment was conducted on fibrillar species formed in α-synuclein:PBSN. Circular dichroism (CD) spectra were obtained for non-PMCA (0 h) and PMCA-generated α-synuclein in both PCB and PBSN. Consistent with the organized rearrangement of α-synuclein upon misfolding, non-PMCA samples of both buffers showed largely random coil present that shifted to become rich in β-sheet structures following PMCA (Fig. 2D). Likewise, using TEM, the morphology of fibrillar species produced in PBSN after PMCA for 72 h (Fig. 2E) was found to be consistent with those produced in PCB (Fig. 1C; Herva et al., 2014). Finally, similar to 24 h α-synuclein:PCB shown in Fig. 1D, 72 h α-synuclein:PBSN was ThT reactive (Fig. 2F). In conclusion, although it cannot be presumed that PBSN precisely mirrors the mechanism of misfolding that occurs using PCB (for example, due to the effect of Triton-X 100 that is present in PCB), collectively these data show that 72 h is an appropriate PMCA process time to produce misfolded species in PBSN that share many similar amyloidogenic and oligomeric properties to protein produced in PCB.

A necessary component to confirming that PBSN is a suitable buffer to produce misfolded α-synuclein that can facilitate studies on its pathogenicity was to confirm that PBSN is non-toxic when applied to cultured cells. To address this, the ability of the buffer alone (void of α-synuclein protein) to cause cell death in neuroblastoma SH-SY5Y cells was measured via the release of lactate dehydrogenase (LDH) into conditioned medium. In order to test the usefulness of PBSN-based buffers in PD model systems, LDH assays were also performed using the A4 cell line (SH-SY5Y cells stably transfected to overexpress wild-type α-synuclein). Prior to LDH measurements, confirmation of elevated α-synuclein expression in A4 cells compared to untransfected SH-SY5Y counterparts came from western immunoblot and immunofluorescence analyses (Fig. S3A,B). Both cell lines were treated with PCB or PBSN such that the total volume of culture medium was composed of 0.5, 1, 5 or 10% v/v buffer and LDH, measured 24 h later (Fig. S3C,E). Results showed a clear dose-dependent increase in the toxicity of PCB (buffer only), with 10% PCB eliciting a near-100% cell death response for SH-SY5Y and A4 cells. In contrast, PBSN (buffer only) showed negligible toxicity at all concentrations tested in both cell lines. The cytotoxicity of the buffers measured by LDH was confirmed by differential interference contrast (DIC) images of cells exposed to PCB or PBSN (Fig. S3D,F).

Collectively, these experiments confirm that PBSN is a suitable buffer to produce α-synuclein species under non-toxic conditions, which greatly improves the capacity of PMCA to be used as a tool to study pathogenic properties of misfolded α-synuclein species. As such, all subsequent experiments using PMCA-generated misfolded α-synuclein were produced using this optimized method.

### PMCA-generated misfolded α-synuclein selectively associates with CA

**Fig. 3.**
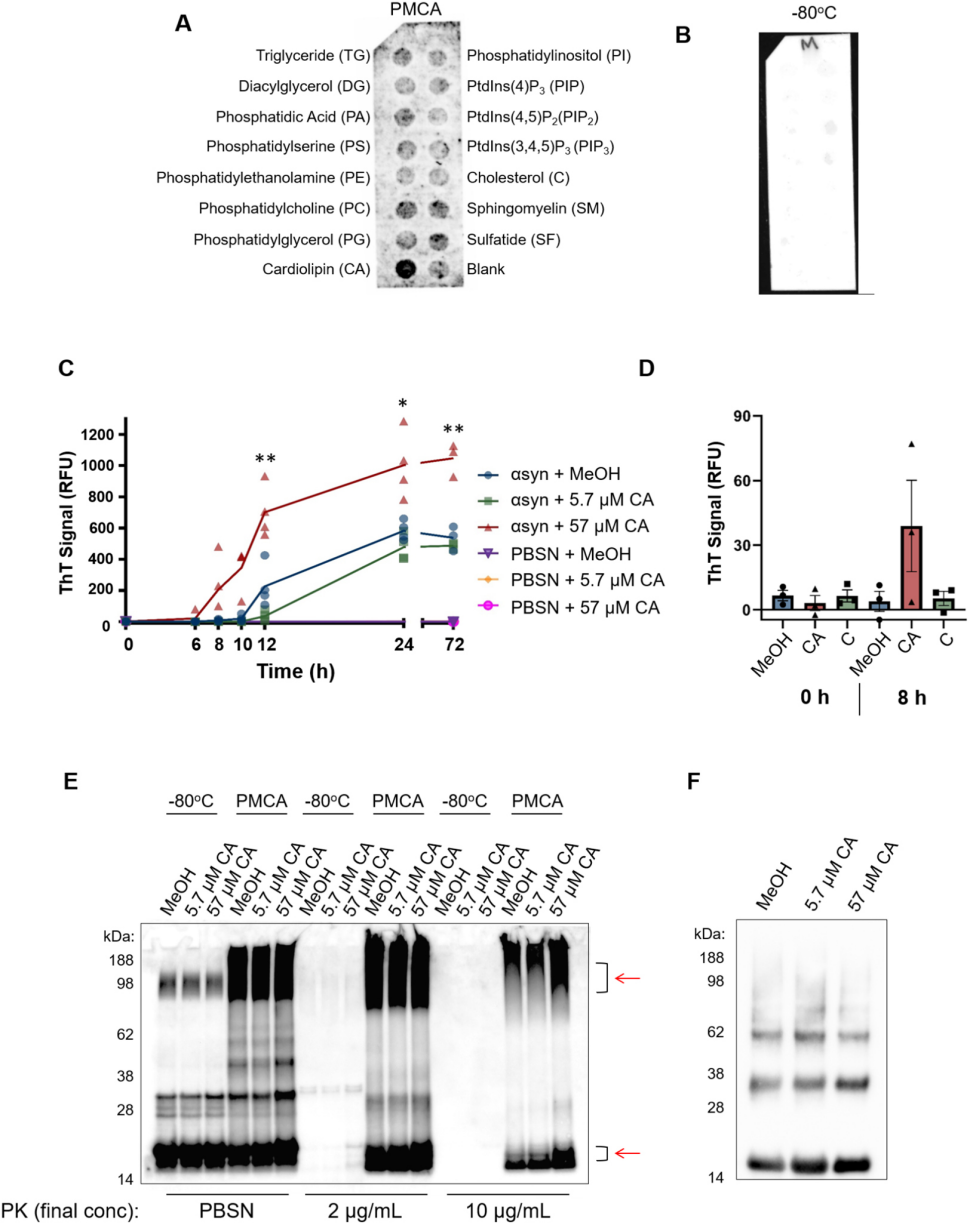
**PMCA-generated α-synuclein selectively associates with cardiolipin.** A hydrophobic membrane strip dotted with 15 different lipids (long-chain >diC16 synthetic analogues) was incubated with PMCA-generated α-synuclein. (A) Binding of α-synuclein to lipids was assessed via western immunoblotting using α-synuclein-specific monoclonal antibody MJFR1 (amino acid specificity: 118–123). (B) Unlike PMCA-generated misfolded α-synuclein, monomeric α-synuclein (−80°C) did not show affinity to any lipid class, *n*=1. To determine whether cardiolipin (CA) modulates α-synuclein fibrillization, CA was dissolved in MeOH/PBSN (MeOH) and added to wild-type α-synuclein to a final concentration of 5.7 μM and 57 µM CA. MeOH/PBSN alone added to α-synuclein:PBSN served to control for the effect of the solvent. (C) Samples were subjected to PMCA for a total process time of 0, 6, 8, 10, 12, 24 and 72 h. Tubes containing PBSN only (no α-synuclein) with solvent or CA exposed to PMCA for 0 h and 72 h served to detect any inherent fluorescence caused by CA or the solvent. Extent of fibrillization was assessed by measuring ThT fluorescence, where the values obtained for each experimental replicate represented the average fluorescence of triplicate wells after subtraction of a blank well to account for background fluorescence. RFU, relative fluorescent units. Data are presented as mean±s.e.m. (*n*=4 for all groups except for 0 h and 72 h, for which *n*=3) and represent every experimental replicate performed. To determine the effect of CA on α-synuclein fibrillization compared to control samples, a mixed-effects ANOVA with Dunnett's multiple comparisons test was employed. *P<0.05, **P<0.01. (D) ThT fluorescence was repeated in the presence of CA, MeOH or cholesterol (C), which showed no binding affinity to α-synuclein in panel A. ThT was measured after 8 h, which represented the earliest detectable rise in ThT by CA observed in panel C. The values obtained for each experimental replicate represented the average fluorescence of triplicate wells after subtraction of a blank well to account for background fluorescence. Data are presented as mean±s.e.m. (*n*=3) and represent every experimental replicate performed. (E) The biochemical properties of α-synuclein species formed after 72 h PMCA (PMCA) or not (−80°C) was assessed by its resistance to proteolysis following digestion in proteinase K (PK) at a final concentration of 0, 2 or 10 µg/ml. PK-resistant species were detected by immunoblotting using MJFR1. Areas of differential PK resistance between PMCA-generated species are identified by red arrows. *n*=1. (F) Ultracentrifugation was performed on α-synuclein in the presence of 5.7 µM and 57 µM CA or buffer control to determine the insoluble load. *n*=1.

The development of a method to produce misfolded α-synuclein under non-toxic conditions using PMCA allowed the commencement of the next aim, which was to identify potential pathogenic mechanisms of misfolded α-synuclein. Although numerous mechanisms of how α-synuclein causes cellular damage have been reported, a common theme unifying several of these mechanisms appears to involve the interaction of misfolded α-synuclein with lipid species (Ugalde et al., 2019). Therefore, to gain insight into potential mechanisms to pursue, the ability of misfolded α-synuclein to interact with various lipids was assessed using a hydrophobic membrane that had been dotted with 15 long-chain (>diC16) highly pure synthetic lipid analogues and a blank control. This membrane was incubated with misfolded (PMCA) or monomeric (−80°C) α-synuclein and the degree of protein binding to the lipids was assessed via immunoblotting using the α-synuclein-specific antibody MJFR1. These qualitative results revealed that, compared to the −80°C sample, PMCA α-synuclein exhibited a general elevation in both lipid-specific and non-specific binding to the membrane (Fig. 3A,B). By far, PMCA α-synuclein exhibited the strongest affinity to CA. Interestingly, the interaction of α-synuclein with these lipid species was conformation specific, with no reactivity with any lipid observed after incubation with the −80°C sample (Fig. 3B). Given that this sample contains monomeric protein and no ordered aggregates, it can be inferred that the proteins interacting with lipids in the PMCA sample are ordered misfolded structures.

The identification of CA as a lipid class with high binding affinity to misfolded α-synuclein using an unbiased screen merited further analysis into whether this lipid can modulate α-synuclein fibrillization. To do this, ThT was employed to study the kinetics of α-synuclein fibrillization in the presence of the lipid. After reconstitution in methanol (MeOH) and PBSN, CA was added to α-synuclein:PBSN to a final concentration of 57 µM and 5.7 µM. MeOH alone was added to tubes with equivalent protein to serve as a control for the effect of the solvent. The ability of CA to modulate α-synuclein fibrillization was determined by measuring ThT fluorescence in individual tubes containing CA exposed to PMCA for a total process time of 0, 6, 8, 10, 12, 24 or 72 h, and analysed compared to equivalent CA-free control samples. Here, compared to tubes containing α-synuclein with MeOH (solvent only), the addition of 57 µM CA accelerated α-synuclein fibrillization, which was evidenced by a statistically significant elevation in ThT first identified after 10 h (*P*=0.031) (Fig. 3C). This elevation was sustained at all subsequent time points (12, 24 and 72 h; *P*=0.009, *P*=0.041 and *P*=0.006, respectively). No significant differences were identified between the control group and the lower concentration of CA (5.7 µM CA). Importantly, neither the lipid nor MeOH exhibited any florescence signal in the absence of α-synuclein (Fig. 3C). In order to determine whether the ability of CA to accelerate α-synuclein fibrillization was confounded by molecular crowding of the lipid, ThT was performed again using CA alongside a lipid found to not associate with the protein, as determined by the lipid strip (Fig. 3A). Here, CA or the equivalent molar concentration (57 µM) of cholesterol (C) was added to α-synuclein and ThT measured after 8 h exposure to PMCA. This time point was chosen to represent the earliest CA-induced elevation in ThT (Fig. 3C) and confirmed that, at this time point, only CA (and not C or buffer controls) exhibited PMCA-induced elevations in ThT (Fig. 3D). No ThT reactivity was observed in any sample at 0 h.

Having observed the ability of CA to modulate α-synuclein fibrillization detected using a fluorescent technique, we sought to complement these observations by examining what effect this has on the misfolded protein produced. It has been shown that species formed by PMCA are more resistant to proteolysis (Herva et al., 2014), which may be a useful assay to detect changes in the population of species formed. Accordingly, the effect of CA on the misfolding of α-synuclein was next assessed by exposing PMCA products to proteinase K (PK) digestion and performing western immunoblotting. PMCA of α-synuclein was performed in reactions containing CA (as per the conditions for Fig. 3C,D) or solvent for 72 h prior to being exposed to 0, 2 or 10 µg/ml PK. These samples were compared to corresponding tubes that did not undergo PMCA (−80°C). Results showed that, in the absence of proteolysis or low concentrations of PK (2 µg/ml), there appeared to be more oligomeric content formed when α-synuclein was incubated with 57 µM CA. Upon treatment with 10 µg/ml PK, an increase in both high- and low-molecular-mass protein was observed in the PMCA sample containing 57 µM CA, compared to all other groups (Fig. 3E). Densitometric analysis revealed that, in the PMCA samples digested with 10 µg/ml PK, the total protein abundance in the sample containing 57 µM CA was 1.38-fold higher than its solvent control (MeOH/PBSN) equivalent. This altered expression appeared to be due to changes in PK-resistant material in the molecular mass range of 90–100 kDa and 15–20 kDa. On the western immunoblot in Fig. 3E, these regions are identified by red arrows. Given that elevated reactivity was observed in both the high- and low-molecular-mass region, these two populations of PK-resistant protein likely reflect the large species that were able to resolve further through the gel following PK digestion and their concomitant cleavage products. Both 2 μg/ml and 10 µg/ml PK digested all protein in all −80°C groups, indicating that neither the CA nor solvent interfered with the activity of PK in this experiment. This assay complements the findings of Fig. 3C and indicates that the acceleration of fibrillization seen is associated with changes to the biochemical properties of formed species in this system. Given that the enhanced proteolysis resistance is potentially related to an increase in fibrillar content, PMCA-generated protein formed in the presence of MeOH (solvent control), 5.7 µM or 57 µM CA was exposed to ultracentrifugation to isolate insoluble protein load. These results showed that CA had a mild influence on increasing insoluble content whereby, compared to buffer control, protein produced in the presence of 5.7 µM and 57 µM CA had an increase in insoluble content of 11% and 13%, respectively (Fig. 3F). Collectively these studies show that PMCA-generated α-synuclein associates with CA, which can influence both the rate of fibrillization and the biochemical and biophysical properties of the species formed.

### PMCA-generated misfolded α-synuclein causes mitochondrial hyperactivity without any functional deficit in naïve SH-SY5Y cells

Within a cell, the expression of CA is almost exclusively localized to the IMM. Hence, the finding that misfolded α-synuclein binds selectively to CA suggests that the mitochondrion is a target for misfolded α-synuclein. A major biochemical process driven by mitochondria is oxidative phosphorylation, which produces the majority of the cell's energy in the form of adenosine triphosphate (ATP). This respiration occurs via the electron transport chain (ETC), which is a series of reactions that take place within the IMM and involves four complexes (I–IV) that collectively act to shuttle electrons between them. This process releases protons into the intermembrane space (IMS), creating a proton gradient between the IMS and the mitochondrial matrix that in turn drives the generation of ATP through the large multi-subunit enzyme complex ATP synthase.

We sought to assess the effect misfolded α-synuclein has on mitochondrial respiration by measuring respiratory flux rates, which are the rates of electron transfer to molecular oxygen via the ETC. This is performed in live cells following the incremental addition of compounds that selectively inhibit components of oxidative phosphorylation. In this regard, information on the functioning of each of the components of oxidative phosphorylation may be obtained. Unlike standard techniques that quantify products of respiration at a single time-point, the ability to measure flux changes in mitochondria is a powerful way to identify both overt and subtle alterations to respiration.

The ability of misfolded α-synuclein to alter mitochondrial respiration was measured in naïve SH-SY5Y neuroblastoma cells using the Seahorse XFe24 Analyzer. In this system, respiration is measured via changes in oxygen (referred to as oxygen consumption rate; OCR) in the medium in wells containing adhered cells. This is performed using a solid-state sensor probe that detects changes in dissolved oxygen in a portion of the medium over time, with multiple readings taken to increase the sensitivity of the assay. Baseline OCR readings were taken from adhered cells prior to the sequential addition of the pharmacological chemicals oligomycin, carbonyl cyanide m-chlorophenyl hydrazone (CCCP), rotenone and antimycin A. Respectively, these compounds inhibit ATP-synthase, permeabilize mitochondrial membranes to protons, inhibit complex I and complex III (in the presence of rotenone this inhibits the flow of electrons from Complex II to oxygen). The data obtained from such measurements allow the following parameters to be measured in a single experiment: basal respiration, ATP synthesis, maximum (max) respiration, Complex I activity, Complex II activity, non-respiratory, proton leak and spare capacity. A schematic showing a typical respirometry experiment is shown in Fig. S4A.

In addition to cells exposed to misfolded α-synuclein, other treatment groups included cells incubated with monomeric α-synuclein (−80°C), buffer alone (PBSN) and cells left untreated (Untreat). The final concentration used was 5% v/v for all treatment groups, which were co-treated with lipofectamine in order to aid the delivery of protein into the cells. Fig. S4B is an example experiment performed in the current study. In these experiments, two treatment groups (PBSN-treated and untreated) were used as controls and hence we first examined whether PBSN alone was causing any inherent changes to mitochondrial respiration compared to untreated cells. As shown in Fig. S4C–H, assessment of mitochondrial readouts found no difference between the two control groups. Because PBSN alone did not contribute to any mitochondrial respiration parameter, these experimental groups were pooled into one group named Control for subsequent assessment compared to monomeric (−80°C) or misfolded (PMCA) protein.

**Fig. 4.**
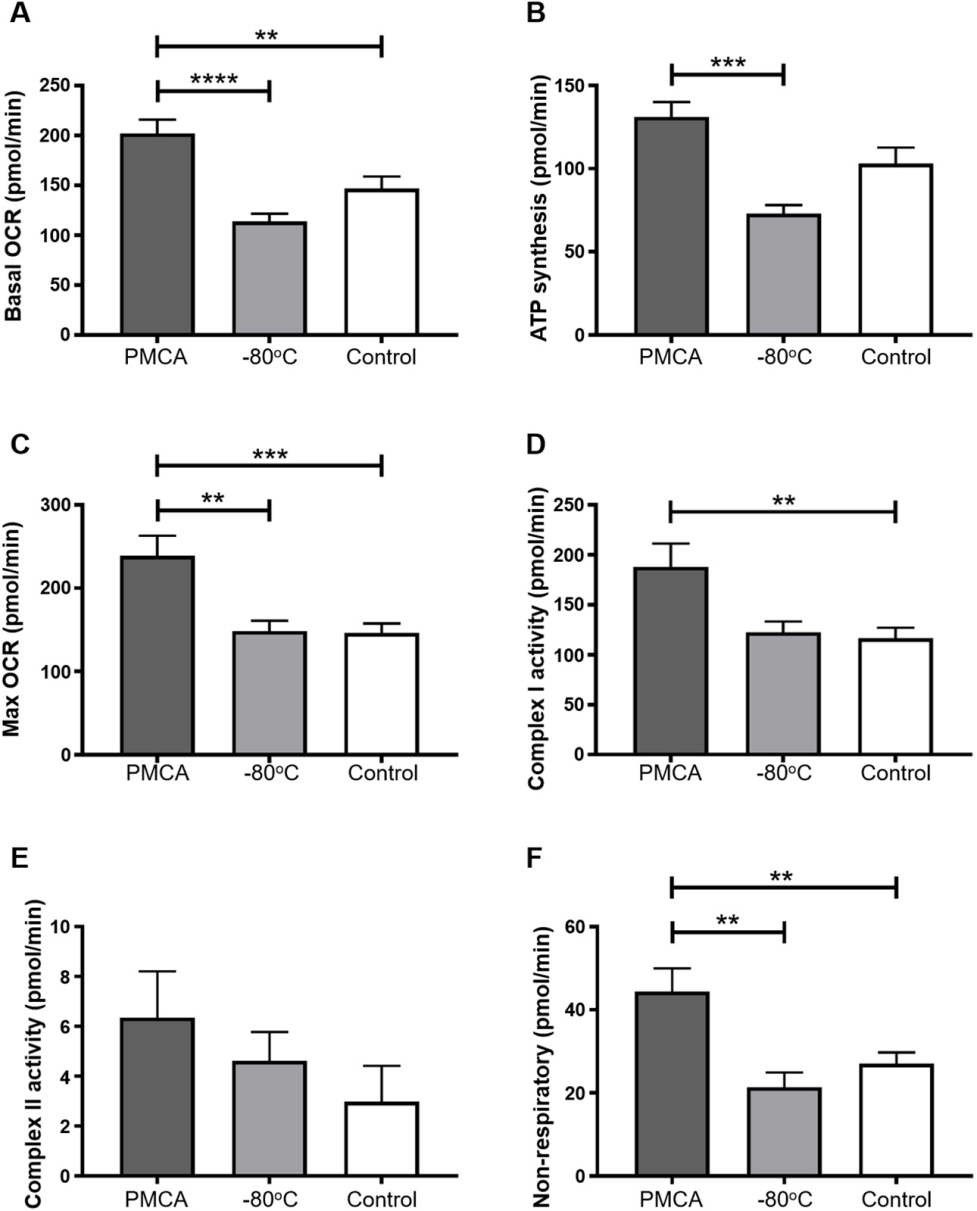
**PMCA-generated misfolded α-synuclein causes mitochondrial respiration to become hyperactive in SH-SY5Y cells*.*** (A–F) SH-SY5Y cells were incubated with PMCA-generated α-synuclein (PMCA), monomeric α-synuclein (−80°C) or buffer alone or left untreated, which collectively represented the control group (Control). Medium-containing cells were plated into each of four wells per sample of a Seahorse XFe24 plate and mitochondria respiration was measured in adhered cells using the Seahorse XFe24 Analyzer. This was performed by detecting changes in oxygen (referred to as the oxygen consumption rate; OCR) following the addition of pharmacological agents: oligomycin, carbonyl cyanide m-chlorophenyl hydrazone (CCCP), rotenone and antimycin A. In doing so, the following parameters were measured: (A) basal OCR, (B) ATP synthesis, (C) max OCR, (D) complex I activity, (E) complex II activity and (F) non-mitochondrial respiration. The average of five wells was taken for each sample per experimental replicate. Data are presented as mean±s.e.m. (*n*=16, 13, 18 for PMCA, −80°C and Control, respectively) and represent every experimental replicate performed. Statistical significance was examined by ANOVA and Tukey's multiple comparisons test with a statistical criterion of 0.01. **P<0.01, ***P<0.001, ****P<0.0001.

Statistical analysis on independent experimental replicates showed that cells exposed to misfolded α-synuclein had an elevated basal OCR compared to both monomeric and control-treated cells (Fig. 4A–F). This significant elevation of OCR in the presence of misfolded α-synuclein was maintained in individual components of mitochondrial respiration: ATP synthesis, max OCR, Complex I activity and non-respiratory (Fig. 4B–D,F, respectively). Importantly, no significant difference was found between −80°C and control groups in any readout. Although Complex II activity was also elevated upon exposure to misfolded α-synuclein compared to the two other groups, this difference did not reach statistical significance in comparisons between any groups (Fig. 4E). However, this observation is not unexpected, given the low overall contribution of Complex II to the OCR in this cell line, relative to the accuracy and reproducibility of the assay. To summarize these data, misfolded α-synuclein causes broad-spectrum elevations in all components of respiration in mitochondria of SH-SY5Y cells. This hyperactivity was specific to misfolded α-synuclein and was not observed when cells were treated with monomeric protein or in control-treated cells. Table S1 lists all significant values from statistical analysis of the cellular respiration data shown in Fig. 4.

**Fig. 5.**
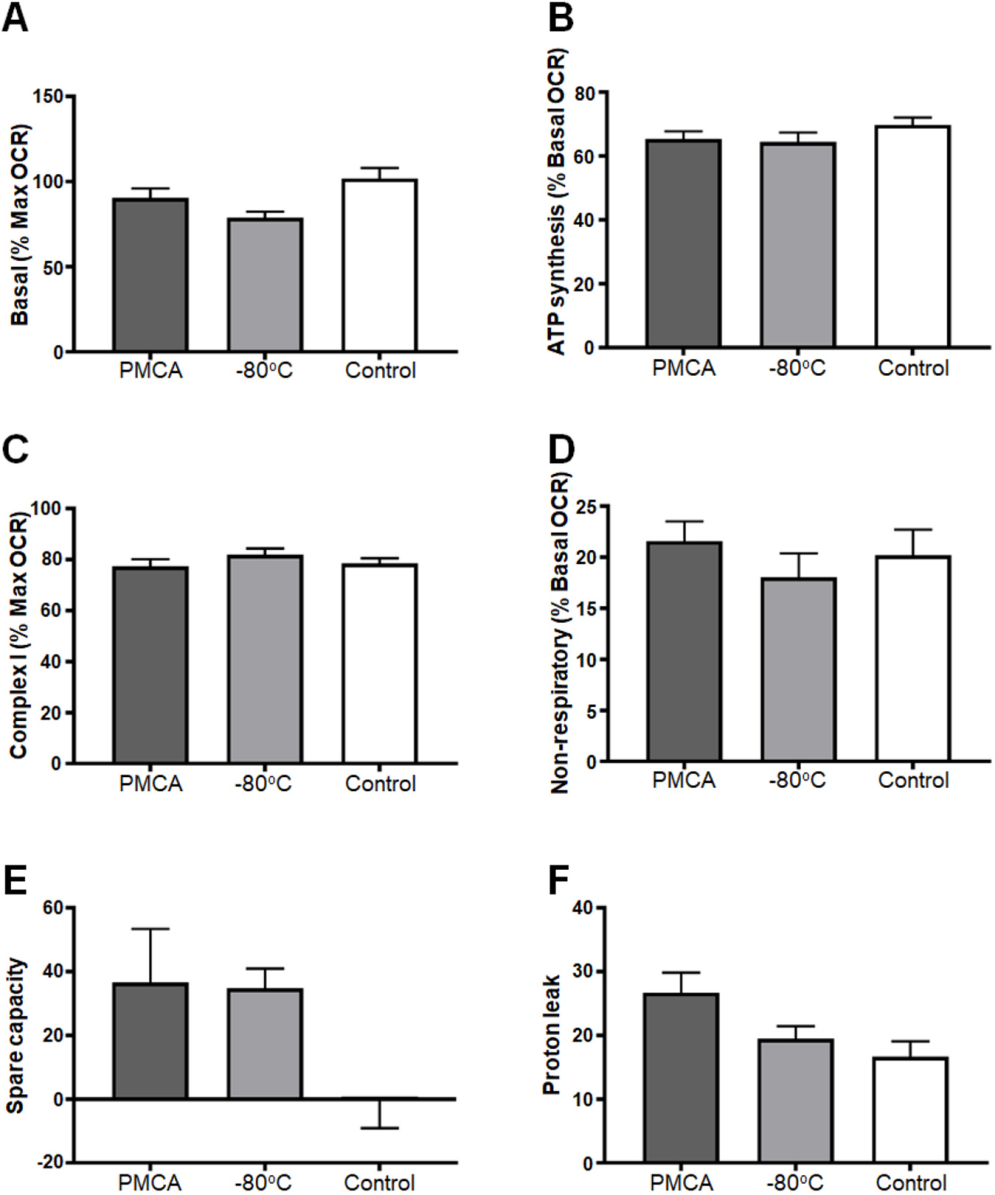
**PMCA-generated misfolded α-synuclein does** **not cause functional deficits in mitochondria of SH-SY5Y cells.** SH-SY5Y cells were incubated with PMCA-generated α-synuclein (PMCA), monomeric α-synuclein (−80°C) or either buffer alone or left untreated, which collectively represented the control group (Control). Medium-containing cells were plated into each of four wells per sample of a Seahorse XFe24 plate pre-coated with Matrigel. The Seahorse XFe24 Analyzer measured mitochondria respiration by detecting changes in oxygen (referred to as the OCR) following the addition of pharmacological agents: oligomycin, CCCP, rotenone and antimycin A. The functioning of mitochondria was determined as parameters expressed as % of max or basal OCR (depending on the functional readout). Spare capacity was measured as the difference between max and basal OCR, and proton leak was measured as the difference between basal and ATP synthesis and non-respiratory. The average of five wells was taken for each sample per experimental replicate. Data are presented as mean±s.e.m. (*n*=16, *n*=13, *n*=18 for groups PMCA, −80°C and Control, respectively) and represent every experimental replicate performed. Statistical significance was examined by ANOVA and Tukey's multiple comparisons test with a statistical criterion of 0.01. No statistical significance was found for all experimental comparisons.

Further analysis of the OCR values obtained in Fig. 4 was performed to determine whether the alterations in respiration identified were associated with concomitant functional deficits. The following functional readouts were calculated: basal (% max OCR), ATP (% basal), Complex I (% max OCR), non-respiratory (% basal OCR), spare capacity and proton leak. No difference was seen in any experimental group (Fig. 5), indicating that misfolded α-synuclein does not affect the function and thus fractional contribution to respiration by any of the individual complexes. Therefore, although misfolded α-synuclein causes mitochondrial hyperactivity, it does not translate to any kind of observable dysfunction in the mitochondria of neuroblastoma SH-SY5Y cells.

### Hyperactivity is not a result of altered glycolysis in SH-SY5Y neuroblastoma cells exposed to misfolded α-synuclein

Having identified the capacity of misfolded α-synuclein to cause hyperactive mitochondrial respiration, we next explored whether other components of cellular respiration were similarly affected. Glycolysis is an important upstream component to cellular respiration; it produces a small amount of the total ATP synthesized by the cell and critically produces acetyl co-enzyme A, which feeds into the tricarboxylic acid cycle, producing the electron donors FADH_2_ and NADH that are essential for oxidative phosphorylation. Given the intimate association between glycolysis and oxidative phosphorylation, the glycolytic potential of SH-SY5Y cells was assessed upon exposure to misfolded α-synuclein, which was measured via the extracellular acidification rate (ECAR) of cells in a glycolytic stress test. Within a cellular environment, ECAR is largely attributed to glycolysis and, similar to the fluxes in cellular respiration, the glycolytic potential of cells may be tested by the sequential addition of compounds: glucose, oligomycin, rotenone + antimycin A and 2-deoxy-D glucose. The combination of these compounds, respectively, provides fuel for glycolysis to occur, inhibits ATP synthase, inhibits electron transport and inhibits glycolysis. The inhibition of electron transport by the combined actions of rotenone and antimycin A allows assessment of any contribution to ECAR by respiratory electron transport. Basic parameters calculated from this included starved ECAR, basal glycolytic ECAR, glycolytic capacity and spare glycolytic capacity. An image depicting a typical experiment measuring glycolytic potential is shown in Fig. S5A. Glycolytic potential of SH-SY5Y cells was measured upon exposure to PMCA-generated misfolded α-synuclein (PMCA), monomeric α-synuclein (−80°C), buffer alone (PBSN) and untreated (Untreat), with the same treatment regime as the mitochondrial respiratory experiments. An example experiment is shown in Fig. S5B and, following three independent experiments, no difference was found between any of the experimental groups in all glycolytic parameters (Fig. S5C–F).

## DISCUSSION

PMCA is an assay shown to be capable of producing a range of various-sized misfolded α-synuclein species (Herva et al., 2014). It was of interest to use this system to study the pathogenicity of misfolded α-synuclein; however, further analysis of the species formed was limited due to a high concentration of detergent in the conversion buffer. Hence, this study had two components: (1) to adapt the published protocol of PMCA-induced α-synuclein misfolding to enable production of α-synuclein to occur under non-toxic conditions; and (2) to use this newly reformed system to investigate pathogenic mechanisms of misfolded α-synuclein. Replication of the original protocol was first achieved, with species produced via PMCA being characterized using a range of readouts (ThT, TEM and western immunoblotting). This confirmed the system as a suitable tool to produce misfolded α-synuclein. The effectiveness of PMCA-induced α-synuclein misfolding when reconstituted in non-toxic buffers was next tested, and adaption of the protocol was performed with the most efficient non-toxic buffer, PBSN. New parameters were determined to produce comparable misfolded content to the initial buffer, with 72 h being the optimized time to misfold α-synuclein in PBSN, as assessed using a range of readouts. The ability of PBSN to be well tolerated in both normal, untransfected and transgenic PD-model neuroblastoma cells was the final assessment in the adaptation of the protocol and allowed further studies into the cellular targets of misfolded α-synuclein.

The major finding of this study was that PMCA-generated α-synuclein species affect mitochondrial respiration. Mitochondrial dysfunction is a central feature of PD, and numerous studies have implicated α-synuclein in decreasing mitochondrial activity in various animal (Bender et al., 2013; Chinta et al., 2010; Li et al., 2013; Martin et al., 2006; Sarafian et al., 2013; Zhu et al., 2011) and cell culture (Devi et al., 2008; Parihar et al., 2008; Reeve et al., 2015) models of disease. In addition to overall decreases in mitochondrial respiration, α-synuclein has been reported to cause functional deficits, particularly at the level of Complex I. Cells expressing wild-type or mutant α-synuclein reportedly have deficits in Complex I (Chinta et al., 2010; Devi et al., 2008), which are also observed in post-mortem PD brain (Janetzky et al., 1994; Parker et al., 1989; Schapira et al., 1989). Hence, the robust hyperactive respiration without functional deficit observed in SH-SY5Y cells in the presence of misfolded α-synuclein reported in the current study is in contrast to the general paradigm that α-synuclein decreases mitochondrial respiration in association with Complex I deficiency.

In order to delineate these divergent findings, it is important to consider factors that may contribute to variations in results. A major influence is likely to be the model used, with cell culture systems, transgenic animal models and human disease all harbouring very different cellular environments. Reporting an acute insult to naïve, healthy cells upon treatment with misfolded protein is likely dissimilar to the disease in humans, which has been slowly progressing over years of clinical dormancy. Similarly, given that the overexpression of protein in cultured cells does not result in intracellular aggregation of protein, it is also unlike the human condition. In this regard, it could be argued that the introduction of misfolded protein into naïve cells is more biologically relevant than a cellular environment in which the clearance machinery is adequately removing protein aggregates. It is important to note that, in the current study, the delivery of α-synuclein into the cell was aided by lipofectamine, which could foreseeably have off-target effects associated with mitochondrial respiration. However, the inclusion of control samples treated with buffer only (PBSN) and lipofectamine showed equivalent readouts to untreated cells devoid of the transfection reagent. Cationic liposomal proteins are commonly used in studies delivering α-synuclein intracellularly (Luk et al., 2009; Nonaka et al., 2010), and we found that its use did not contribute to mitochondrial readouts. Regardless, while each system identifies an effect of α-synuclein, its relevance to *in vivo* PD is uncertain. However, this also reflects an important point with data collected on human samples, which report the status of mitochondria at post-mortem and hence terminal stages of disease. Detailing this stage of disease, while relevant, may not be an adequate representation of mitochondrial health for the majority of the disease process. Considering this, it is possible that the mitochondria are able to adapt to the insult caused by α-synuclein for the majority of the disease course, before being overwhelmed at end-stage disease. Such a scenario would align with the findings of this study. In support of this, numerous studies on mitochondrial respiration in various cell types in small cohorts of ante-mortem PD patients have failed to produce conclusive findings (Ambrosi et al., 2014; Barroso et al., 1993; Bravi et al., 1992; Cronin-Furman et al., 2013; Esteves et al., 2010; Martin et al., 1996; Parker and Swerdlow, 1998; Shinde and Pasupathy, 2006; Yoshino et al., 1992). Importantly, recently, a larger study examining mitochondrial respiration in immortalized lymphocytes from 30 patients found enhanced respiratory activity with no functional deficit (Annesley et al., 2016). This elevation was found to be independent of age, disease duration or disease severity and hence supports the theory that mitochondria are adaptive in disease. Collectively, while further assessment is merited in models of disease, the data presented here using misfolded α-synuclein in non-transgenic cells suggest that the dogma that mitochondria dysfunction is a persistent, overwhelming feature of PD may ultimately be revised.

Given that PMCA produces a heterogeneous population of α-synuclein species, this work did not distinguish which species of misfolded α-synuclein is causing the changes to mitochondrial respiration. An attractive candidate is the oligomeric conformations, given that these species have been shown to fragment CA-rich membranes (Nakamura et al., 2011); however, other species may be relevant. In transgenic mice overexpressing wild-type α-synuclein, the predominant species that accumulated in the mitochondria causing deficits was monomeric full-length N-terminally acetylated α-synuclein (Sarafian et al., 2013). The latter study highlights an important consideration when using recombinant protein to study pathogenic mechanisms of α-synuclein, which are not exposed to the numerous post-translational modifications endogenous α-synuclein is susceptible to. In PD, disease-associated α-synuclein is largely phosphorylated at position Ser129 (pSer129) (Fujiwara et al., 2002), and, in cultured cells expressing mutant A53T α-synuclein, both the stimulation of reactive oxygen species production and alterations to mitochondrial respiration highly correlate with levels of pSer129 (Perfeito et al., 2014). Therefore, the production of misfolded species using PMCA may not model all pathogenic subtypes of α-synuclein that are present in a cell and cause damage in disease. An additional point is the buffer used in this study. High salt concentrations have been shown to modulate α-synuclein misfolding and hence further assessment will be necessary to examine the similarities between PMCA-generated α-synuclein produced in PBSN and protein misfolding that occurs *in vivo*.

This work did not elucidate whether the changes in mitochondrial respiratory function upon exposure to misfolded α-synuclein were due to a direct or indirect mechanism. The observation in Fig. 3A that PMCA-generated α-synuclein species associate with CA is support for α-synuclein modulating mitochondria respiration via a direct interaction with this lipid. CA is commonly referred to as the signature phospholipid of the mitochondrion. It is predominately expressed in the IMM, which contains more than 40-fold higher levels of CA than the outer mitochondrial membrane (de Kroon et al., 1997) and is a component of and/or required for the optimal functioning for all major components of the ETC (Paradies et al., 2014). Therefore, consistent with the elevated mitochondrial oxidative phosphorylation reported in this study, if an interaction between α-synuclein and CA occurs in a biological setting, it would likely result in broad-spectrum changes to respiration. Further support for a direct interaction of α-synuclein with mitochondria comes from many studies showing that mutant or wild-type α-synuclein associates with isolated mitochondria (Nakamura et al., 2008; Parihar et al., 2008) and mitochondria *in vivo* (Chinta et al., 2010; Cole et al., 2008; Devi et al., 2008; Parihar et al., 2008; Shavali et al., 2008). Also, α-synuclein has been shown to bind to membranes mimicking mitochondrial membranes, with a preference for those containing CA (Nakamura et al., 2011; Zigoneanu et al., 2012).

Given that this current work does not confirm a direct interaction between misfolded α-synuclein and mitochondria, it is possible that indirect mechanisms are driving the enhanced mitochondrial respiratory activity. As a general rule, enhanced respiration to all complexes without altering function would implicate an indirect mechanism. The most likely candidates for this are those that trigger an increase in ATP production. Autophagic flux is a major modulator of cellular respiration and thus the detection and clearance of misfolded α-synuclein may be a major contributing pathway to the changes in respiration seen here. However, the finding that glycolysis was unaffected upon exposure to PMCA-generated α-synuclein reveals important information on the potential mechanism of action. It suggests that if mitochondrial respiration was enhanced because of increased energy demands following exposure to misfolded α-synuclein, it did not also drive changes in glycolysis. This is consistent with the fact that no mitochondrial deficits were identified and hence glycolysis was not upregulated as a compensatory mechanism. It also eliminates changes to glycolysis as a driver of the effects seen. It should be borne in mind that both the glycolysis and mitochondrial function assays used here were designed to avoid constraints on activity by substrate supply rates. Furthermore, while further analysis is merited, observing hyperactive mitochondrial respiration following acute exposure of α-synuclein in this system is likely independent of chronic adaptive pathways.

In conclusion, the aim of this study was to develop a system to produce a heterogeneous population of misfolded α-synuclein species under non-toxic conditions and to investigate pathogenic mechanisms associated with these species. We show the development and characterization of a protocol to generate α-synuclein species in the non-toxic buffer PBSN using PMCA. These misfolded species were found to associate with the mitochondrial-specific lipid CA. CA accelerates α-synuclein fibrillization and alters the formation of α-synuclein species produced by PMCA. Critically, PMCA-generated misfolded α-synuclein was shown to enhance mitochondrial respiratory activity in neuroblastoma SH-SY5Y cells. While it is important to note that there are considerations with the work presented in the current study with regards to modelling disease-associated α-synuclein, the findings provide evidence that this mechanism can be a feature of misfolded α-synuclein and, hence, highlight the need for further assessment on the relationship between α-synuclein and mitochondria. The findings of this study provide important insight into pathogenic features of α-synuclein, which may ultimately have implications for our understanding of the pathogenesis of synucleinopathies, including PD.

## MATERIALS AND METHODS

### Preparation of recombinant protein for α-synuclein PMCA

The open-reading frame of the wild-type human α-synuclein gene *SNCA* had previously been cloned into the plasmid pRSET B under the control of the T7 promotor (Cappai et al., 2005). Recombinant human α-synuclein produced using this plasmid was purchased from Monash Protein Production Unit (Monash University, Clayton, VIC, Australia) and supplied as 1-ml aliquots in distilled water (dH_2_O). Purchased protein was lyophilized overnight using a benchtop freeze dry system (Labcono, USA) and stored at −80°C until reconstitution in buffer. The following buffers were used in this study: PCB [1% (v/v) Triton-X 100, 150 mM NaCl, cOmplete ULTRA protease inhibitor cocktail tablet (1 per 10 ml; Roche)/phosphate buffered saline (PBS; Amresco, Solon, OH, USA)], PBS, PBS+150 mM NaCl (PBSN), Tris buffered saline (TBS; Amresco, Solon, OH, USA) and TBS+150 mM NaCl (TBSN). The final NaCl composition of buffers was 0.137 M, which increased to 0.287 M for those buffers with the additional 150 mM NaCl. For reconstitution of protein, 10–16 mg protein was dissolved in 1 ml buffer and centrifuged at 122,500 ***g*** for 30 min, 4°C (Optima™ MAX Ultracentrifuge, Beckman Coulter) to sediment amorphous aggregates. Protein concentration of the supernatant was determined by measuring protein absorbance at 280 nm using a photometer (BioPhotometer, Eppendorf) and employing the Beer-Lambert law. Protein was diluted to a final concentration of 90 μM and 60 μl aliquoted into PCR tubes and stored at −80°C until experimental use in α-synuclein PMCA.

### α-Synuclein PMCA

α-Synuclein PMCA was performed as described previously (Herva et al., 2014). PCR tubes containing α-synuclein were briefly spun in a benchtop centrifuge (Microfuge^®^16 centrifuge, Beckman Coulter), 37±3 mg beads (1.0 mm zirconia/silica beads, BioSpec Products) were added and tubes were sealed with Parafilm (Pechiney Plastic Packaging Company, Chicago, IL, USA). Samples were placed into a 96-tube rack in a microplate cup-horn sonicator (Misonix 4000) that was filled with water using a circulating water bath to maintain a steady-state temperature of 37°C. PMCA reactions using α-synuclein consisted of 20 s of sonication every 29 min 40 s for varying lengths of time. Power setting 7 was used for all α-synuclein PMCA experiments. For protease resistance studies, PK was diluted in PBSN to 6× the desired final concentration and 4 μl was added to 20 μl of the PMCA product prior to incubation at 37°C for 30 min with occasional agitation. Replacement of PK with PBSN in equivalent samples acted as non-PK controls and these samples were kept at room temperature (RT) during the digestion of PK-containing samples. All samples were then diluted in 4× NuPAGE LDS sample buffer (Life Technologies) and boiled at 100°C for 10 min. Samples were resolved using SDS-PAGE and western immunoblotting was employed to detect α-synuclein species using the antibody MJFR1 (ab138501, Abcam, Cambridge, UK; 1:10,000 dilution) (Brudek et al., 2016). To assess insoluble load of protein exposed to PMCA, ultracentrifugation was used. Following a PMCA, the reaction sample was diluted in PBSN to fill a 200-μl-capacity thick-walled polyallomer tube (Beckman-Coulter). Samples were spun at 436,000 ***g*** for 1 h at RT. The pellet containing insoluble protein was washed in PBSN and centrifuged again under the same conditions. The supernatant was then discarded and the pellet resuspended in 8 M urea/PBSN and incubated for 30 min at RT. Western immunoblot analysis to detect human α-synuclein was performed as described previously.

### ThT assay

Extent of fibrillization was quantified in PMCA products by reactivity to ThT (Sigma-Aldrich). Here, protein solution was diluted 1/25 in ThT solution (20 μM ThT, 50 mM glycine in dH_2_O, pH 8.5 with KOH) to a final volume of 250 μl in a well of a 96-well black polystyrene plate (Nunc™, Thermo Fisher Scientific). Fluorescence was measured using a Varioskan plate reader (Thermo Fisher Scientific). Triplicate wells of the same sample were averaged and values determined following subtraction of recordings of ThT solution void of sample to account for background fluorescence. When determining the effect of a compound on PMCA-induced α-synuclein fibrillization using ThT, separate tubes containing the compound in 60 μl PBSN only were added to the PMCA for 72 h, as well as protein containing the compound left at −80°C for the duration of the PMCA. These served as controls to confirm that no ThT signal was attributed to the compound in the presence and absence of PMCA. For studies investigating kinetics of α-synuclein fibrillization, individual PCR tubes of α-synuclein were placed into the 96-tube rack of a running sonicator that was continuously cycling. This was done at specified intervals to enable all tubes to be removed at the same time, such that their total process time corresponded to the time point of interest.

### TEM

For imaging PMCA products using TEM, samples were applied to formvar-coated grids and visualized by negative staining with uranyl acetate (2% w/v in H_2_O), with images captured using a Joel JEM-2100 electron microscope.

### CD spectroscopy

Conformational changes in α-synuclein were investigated using CD spectroscopy essentially as described previously (Peverelli et al., 2016). Briefly, spectra were acquired using an Aviv Biomedical Model 420 CD spectrophotometer at 25°C using 1 mm quartz cuvettes over a spectral range of 190–260 nm, a step size of 0.5 nm, an averaging time of 4 s and a slit bandwidth of 1 nm. Measurements for each sample or buffer were taken in triplicate, averaged and reported as mean±s.d. Prior to measurements, α-synuclein samples (in PBSN or PCB buffer) were diluted in ultrapure water to a final protein concentration of 100 µg L-1 or 65 µg L-1 for PMCA- and PCB-treated samples, respectively. Readings were taken on α-synuclein protein either exposed to PMCA in PBSN (72 h), PCB (24 h) or left at −80°C (0 h). Spectra for buffers alone, diluted equivalently in ultrapure water and averaged from triplicate measurements, were subtracted from protein spectra in all cases. Protein spectra are transformed into units of Δε (Kelly et al., 2005) to account for differences in protein concentration between samples.

### AUC

AUC sedimentation velocity experiments were performed using a Beckman Coulter XL-A analytical ultracentrifuge equipped with absorption optics and using either a four-hole An60 Ti or eight-hole An50 Ti rotor essentially as described previously (Gupta et al., 2018; Peverelli et al., 2016). Briefly, two-channel epon centrepiece quartz cells were loaded with 300–380 µl α-synuclein (initial protein concentration of 1.3 mg/ml) and 320–400 µl reference buffer (PBSN). Data were collected at either 25°C or 37°C continuously in absorbance mode using a wavelength of 232 nm, rotor speed of 42,000 rpm, radial distance of 6–7.3 cm and radial step size of 0.003 cm without averaging, and for up to 300 scans. An initial radial scan at a lower rotor speed of 3000 rpm was additionally collected for PMCA-generated α-synuclein samples. The partial specific volume of α-synuclein (0.735 ml/g and 0.740 ml/g at 25°C and 37°C, respectively), solvent density and viscosity were calculated as appropriate to the experiment temperature using the software SEDNTERP (available from http://jphilo.mailway.com). Multiple time-staggered sedimentation velocity scans were fitted to *c*(*M*) distribution models using the software SEDFIT v16.1c (Schuck, 2000; Schuck et al., 2002). During fitting, the weight-average frictional ratio (*f*/*f*_0_), meniscus and bottom radial positions were treated as floating parameters. Data were corrected for time-invariant noise, models solved to a resolution of 250 and maximum entropy regularization used (*P*=0.68).

### Lipid binding assays

Lipid strips dotted with 15 long-chain (>diC16) highly pure synthetic lipid analogues were purchased from Echelon Inc. and used to determine the affinity of PMCA-generated misfolded or −80°C monomeric (non-PMCA) α-synuclein to lipid species. Strips were blocked in 2% skim milk (2 h, RT), prior to incubation with 10 μg/ml α-synuclein protein diluted in blocking buffer for 2 h at RT. The extent of binding was measured using immunoblotting by detection of α-synuclein using MJFR1 antibody.

### Preparation of lipids

First, 500 μg synthetic 16:0 CA (Echelon, Inc.) was diluted in a small volume of MeOH and solubilized by sonication (Misonix 3000) at 45°C for 10 min. Cholesterol (Ajax Chemicals) was solubilized in a small volume of chloroform:MeOH (2:1), air dried and resuspended in MeOH. Lipid/MeOH was diluted to a final concentration of 741 µM or 74.1 µM, and 5 μl was added to PCR tubes containing 60 µl of 90 µM α-synuclein dissolved in PBSN.

### Transfection of SH-SY5Y cells to produce stable cell lines overexpressing α-synuclein

In this study, all experiments with cultured cells used SH-SY5Y neuroblastoma cells that were confirmed mycoplasma negative, obtained from American Type Culture Collection. To stably overexpress wild-type α-synuclein in SH-SY5Y cells, the α-synuclein:pcDNA3+ plasmid was used. SH-SY5Y cells were seeded in complete medium [Dulbecco's modified Eagle medium (DMEM) supplemented with 10% (v/v) fetal calf serum, 1% GlutaMAX (v/v) and 1% penicillin/streptomycin 100× (v/v); Life Technologies] in a 12-well tissue culture plate at a density of 1.5×10^5^ for use 48 h later. Cells were serum starved in 750 μl serum-free medium for 4 h prior to transfection. Varying amounts of plasmid and lipofectamine transfection reagent (Thermo Fisher Scientific) were combined separately in 125 μl Opti-MEM (Thermo Fisher Scientific) per well, before being combined, mixed well and left to incubate for 20 min at RT. Then, 250 μl of the combined solution was added dropwise to each well and incubated in 37°C, 5% CO_2_ for 6 h before being replaced with fresh complete medium. Cells were left to recover for 48 h before addition of the selective antibiotic G418 (300 μg/ml; Thermo Fisher Scientific) into complete medium without added penicillin/streptomycin. Expression of α-synuclein in clones derived from transfected single-cell colonies was monitored by western immunoblot analysis, with the cell line expressing the highest level of α-synuclein used for further experiments. The α-synuclein-overexpressing SH-SY5Y cell line used in this study was derived from clone A4 and hence named as such.

### LDH cytotoxicity assay

An LDH cytotoxicity assay kit (Thermo Fisher Scientific) was used to determine the cytotoxicity of buffers used in PMCA to A4 and untransfected SH-SY5Y cells. The protocol followed was as per the manufacturer's instructions. Briefly, 1×10^4^ cells were seeded into a 96-well tissue culture plate and, 24 h later, triplicate wells were treated with 10 μl PBSN or PCB diluted in dH_2_O to achieve a final concentration of 0.5, 1, 5 or 10% final volume of buffer. Additionally, one set of triplicate wells was treated with dH_2_O to act as a control for spontaneous cell death and one set was left untreated to be treated later with lysis buffer to measure maximum cell death (maximum LDH activity). Cells were incubated under standard cell culture conditions for 24 h. Following incubation, 10 μl lysis buffer was added to the set of untreated triplicate wells and incubated again for a further 45 min. Then, 50 μl of medium from all wells was transferred to a 96-well flat-bottom plate. Reaction mixture (50 μl) was added to each well and incubated for 30 min at RT protected from light, followed by the addition of 50 μl stop solution. Absorbance was measured at 490 nm and 680 nm to obtain sample fluorescence and background fluorescence, respectively. To determine LDH activity, the 680 nm absorbance was subtracted from the 490 nm value. Percentage cytotoxicity of a sample was determined using the formula: 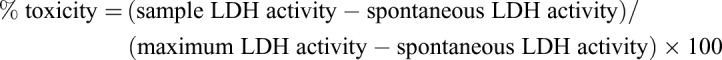


### Western immunoblot analysis of cell lysates

All steps to lyse cultured cells were performed on ice using pre-chilled reagents. Pelleted cells were washed twice with cold PBS and resuspended in lysis buffer [150 mM NaCl, 50 mM Tris pH 7.4, 1% (v/v) Triton X-100, 1% (w/v) sodium deoxycholate (Sigma-Aldrich) and cOmplete ULTRA protease inhibitor in dH_2_O]. Cells were left to lyse for 20 min with occasional agitation, then unwanted debris and nuclear material was removed by centrifugation (1000 ***g***, 3 min). The supernatant was collected and either used immediately in protein determination and western immunoblot analyses, or stored at −80°C for future use. Immunodetection of α-synuclein was performed using MJFR1 antibody.

### Detection of α-synuclein in transfected SH-SY5Y cells using immunofluorescence

A4 and untransfected SH-SY5Y cells (3×10^4^) were seeded into eight-well Lab-Tek^®^II Chamber Slides (Nalge Nunc, Naperville, IL, USA) and left for 48 h to strongly adhere. Cells were washed once in PBS and fixed with 4% (w/v) paraformaldehyde (PFA; Sigma-Aldrich) in PBS for 15 min at RT. Cells were washed three times in PBS to remove residual PFA and permeabilized in 0.5% (v/v) Triton X-100/PBS for 2 min, RT. Cells were washed again as described above and blocked in buffer [2% (w/v) bovine serum albumin (BSA; Sigma-Aldrich)/PBS] for 30 min at RT. Blocking buffer was removed and cells were incubated with primary antibody (MJFR1 to detect α-synuclein) diluted in blocking buffer for 2 h at RT, followed by five washes in PBS. Fluorophore-conjugated (anti-rabbit, Alexa Fluor 568-conjugated) secondary antibody and 4′,6-diamidino-2-phenylindole (DAPI), also diluted in blocking buffer were then added to the chamber wells and left for 2 h at RT before a final five washes in PBS. Walls of the chamber slide were removed and mounted with glass coverslips (Mediglass, Taren Point, NSW, Australia) with ProLong™ Gold Antifade Mountant (Thermo Fisher Scientific). Slides were inverted and left to dry overnight in the dark before being sealed with nail polish (Sally Hansen). Images were taken on a SP5 (Leica Microsystems) confocal microscope.

### Preparation of samples for measuring cellular readouts using the Seahorse XFe24 Analyzer

When preparing α-synuclein and buffer control treatment samples for experiments using the Seahorse XFe24 Analyzer, autoclaved beads were used, and PBSN samples were generated by adding buffer (60 µl) to PCR tubes along with beads (equivalent to the setup with PMCA-generated misfolded α-synuclein), and were similarly exposed to PMCA for 72 h. Confluent SH-SY5Y cells grown in T75 flasks were harvested, pelleted and resuspended in XF assay medium (unbuffered DMEM supplemented with 2.5 mM glucose and 1 mM sodium pyruvate). PMCA-generated misfolded and monomeric (−80°C) α-synuclein protein or PBSN only (26.25 μl per well) was mixed with lipofectamine (3 μl per well) and the combined solution added to the cells (2×10^5^ cells per well) in suspension and mixed gently. Equivalent cell densities were similarly left untreated. Cells and protein (525 μl in total) were then plated into the well of a Seahorse XFe24 plate that had been pre-coated (and subsequently air dried) with Matrigel (356231, Corning) diluted 1:2 in XF assay medium. Cells were left to adhere for 1 h at 37°C prior to analysis with the Seahorse XFe24 Analyzer with readouts calculated as per 200,000 cells.

### Measuring mitochondrial respiratory function in SH-SY5Y cells

Mitochondrial respiratory function in live SH-SY5Y cells was measured using the Seahorse XFe24 Extracellular Flux Analyzer (Seahorse Bioscience) via changes in the OCR following the sequential addition of pharmacological agents (2 µM oligomycin, 1 µM CCCP, 5 µM rotenone, 1 µM antimycin A). Between each compound treatment, the average of three measurement cycles of OCR was taken, each cycle including a 3-min mix step, a 2-min wait and a measurement time of 3 min. Each condition tested had a minimum of three replicate wells, with the average of the values taken per experiment. Equipment setup for glycolysis measurements was as per respiratory function experiments, and glycolytic function was determined from changes in pH associated with the ECAR in live SH-SY5Y cells. This was measured following the successive addition of pharmacological agents (10 mM glucose, 2 µM oligomycin, 5 µM rotenone 5 µM + antimycin A, 100 mM 2-deoxy-D glucose).

### Data analysis

Densitometric analysis was performed on unsaturated western immunoblot images using Image Lab. All statistical analysis was performed using GraphPad Prism. A statistical criterion of 0.05 was used for all experiments except for mitochondrial respiration data, which used 0.01. Normality was assessed on all samples subjected to statistical analysis to ensure that data met the assumptions of the tests used and statistical outliers identified. When values from at least three independent replicates were combined, they were depicted as mean± s.e.m.

## Supplementary Material

Supplementary information
